# Tribocorrosion Mechanisms of Inconel^®^ 718 Under Intermittent Sliding in Alkaline Environment

**DOI:** 10.3390/ma19183962

**Published:** 2026-09-18

**Authors:** Marcin Kowalski, Arkadiusz Stachowiak, Dariusz Ulbrich, Piotr Banas, Konrad Gruber, Dariusz Bartkowski, Aneta Bartkowska

**Affiliations:** 1 Faculty of Civil Engineering, Mechanics and Petrochemistry, Warsaw University of Technology Branch in Płock, Łukasiewicza 17, 09-400 Płock, Poland; marcin.kowalski2@pw.edu.pl; 2 Faculty of Civil and Transport Engineering, Poznan University of Technology, Jacka Rychlewskiego 1, 61-131 Poznan, Poland; arkadiusz.stachowiak@put.poznan.pl (A.S.); piotrkacperbanas@gmail.com (P.B.); 3 Faculty of Mechanical Engineering, Wroclaw University of Science and Technology, Łukasiewicza 5, 50-371 Wrocław, Poland; konrad.gruber@pwr.edu.pl; 4 Faculty of Mechanical Engineering, Poznan University of Technology, Piotrowo 3, 61-138 Poznan, Poland; dariusz.bartkowski@put.poznan.pl; 5 Faculty of Materials Engineering and Technical Physics, Poznan University of Technology, Jana Pawła II 24, 61-138 Poznan, Poland; aneta.bartkowska@put.poznan.pl

**Keywords:** tribocorrosion, Inconel 718, additive manufacturing, cast, alkaline environment

## Abstract

**Highlights:**

- The applied heat treatment increased the hardness of all investigated Inconel 718 variants.
- Higher hardness values were observed for the additively manufactured materials compared with their conventionally manufactured samples.
- All investigated Inconel 718 variants exhibited the ability to passivate in the borate solution.
- Under tribocorrosion conditions, lower material loss was observed for the additively manufactured samples.
- For all investigated material variants, wear under tribocorrosion conditions was consistently higher than that observed under mechanically dominated wear loading.

**Abstract:**

Inconel 718 (IN718) is a nickel-based superalloy known for its outstanding resistance to both wear and corrosion, making it widely used in numerous industrial sectors. The primary objective of this study was to perform a comparative evaluation of the tribocorrosion behavior of conventionally manufactured and additively manufactured Inconel 718 alloys in an alkaline environment, both in the as-manufactured and heat-treated conditions. Tribocorrosion tests were conducted using a ball-on-plate tribological stand. During the experiments, both the coefficient of friction and electrochemical current were continuously monitored, while the resulting wear was evaluated both quantitatively and qualitatively. Under tribocorrosion conditions, lower material loss was observed for the additively manufactured samples. The volumetric wear of the additively manufactured alloy decreased from 0.888 × 10^−3^ mm^3^ in the manufactured condition to 0.719 × 10^−3^ mm^3^ after heat treatment, whereas for the conventionally manufactured alloy the wear volume decreased from 0.906 × 10^−3^ mm^3^ to 0.772 × 10^−3^ mm^3^ after heat treatment. The results indicate that heat treatment reduced material loss under tribocorrosion conditions for both manufacturing methods (about 15–19%). The presented analysis also showed that the manufacturing method did not affect the volumetric wear under tribocorrosion conditions.

## 1. Introduction

Inconel 718 is a nickel-based superalloy containing more than 50% nickel, widely recognized for its excellent mechanical strength, high oxidation resistance at elevated temperatures, corrosion resistance, resistance to cyclic fatigue loading, and good weldability [1,2,3]. Owing to this unique combination of properties, the alloy has become one of the most important engineering materials used in demanding applications, particularly in the aerospace, space, cutting tools and nuclear industries [4,5,6]. Inconel 718 can be produced using a variety of manufacturing technologies, ranging from conventional methods to advanced additive manufacturing processes. In additive manufacturing (AM), components are fabricated layer by layer through the deposition and fusion of metal feedstock, enabling a high degree of control over the final geometry [7,8]. The dominant metal processes are powder bed fusion with a laser (PBF-LB/M) or electron beam (PBF-EB/M) and directed energy deposition using a laser (DED-LB/M), electron beam (DED-EB/M), or arc (DED-Arc/M) source (see ISO/ASTM 52900:2021 [9]). Compared with conventional manufacturing methods, this approach offers the ability to produce complex shapes with reduced material consumption and lower waste generation [10].

Inconel 718, both conventionally manufactured and additively produced, was extensively investigated in a wide range of research areas, including microstructural characterization [11,12], evaluation of fundamental material properties [3,13,14], and assessment of its performance under extreme temperature conditions [15]. Considerable attention was also devoted to investigating the wear resistance [16,17], corrosion behavior [18], and tribocorrosion performance of the alloy in various environments [10,19,20]. In addition to evaluating the factors contributing to material degradation, numerous studies focused on identifying and understanding the wear mechanisms [21,22,23] responsible for the deterioration of Inconel 718 under different service conditions [24,25].

In addition, research was conducted to compare the properties of IN718 produced using different manufacturing methods. Zhao et al. [26] investigated the microstructure and mechanical properties of IN718 superalloys fabricated by PBF-LB/M and conventional casting. Their results showed that the PBF-LB/M-produced IN718 exhibited superior mechanical properties and a more refined microstructure compared with conventionally manufactured IN718. Furthermore, Choi et al. [27] demonstrated that the scanning speed applied during the PBF-LB/M process significantly affected the microstructure and mechanical properties of IN718 components. The properties of IN718 can be modified using various post-processing methods, such as boriding [14,28], hot isostatic pressing combined with heat treatment (HIP + HT) [29,30,31], and heat treatment (HT) [32,33]. These processing methods alter the microstructure, microhardness, and wear resistance of the alloy.

Despite the extensive research conducted on nickel-based alloys, relatively few studies investigated their resistance to the simultaneous action of wear and corrosion (tribocorrosion), especially in the case of additively manufactured materials such as IN718. Song et al. [34] demonstrated that the localized tribocorrosion behavior of Inconel 625 coatings exhibited significant heterogeneity. The formation of macroscopic galvanic cells accelerated corrosion in worn areas and promoted the development of distinct cathodic protection zones surrounding the wear tracks. The environment to which the alloy is exposed plays a significant role in tribocorrosion behavior. For Inconel 625, a higher coefficient of friction was reported in HNO_3_ than in H_2_SO_4_ solution. Moreover, the oxidizing nature of HNO_3_ promoted the formation of multiple oxide layers and intensified tribocorrosion damage [35]. Ho et al. [19] demonstrated that, for additively manufactured IN718, an increase in polarization potential resulted in higher corrosion rates, increased coefficients of friction, and greater material loss due to wear. The overall degradation under tribocorrosion conditions was nearly twice that observed under mechanical alone, indicating a strong synergy between electrochemical and mechanical effects [11]. Other studies on the tribocorrosion behavior of additively manufactured IN718 showed that the corrosive environment increased sample wear by less than 30% [36]. Also, the corrosion ratio can reduce plastic capacity and ultimate capacity [37]. However, these findings do not explain how heat treatment affects the friction and wear resistance of additively manufactured IN718 under corrosive conditions. Furthermore, comparisons between the tribocorrosion performance of conventionally manufactured IN718 and IN718 produced by additive manufacturing in an alkaline environment remain limited.

The primary objective of the present study was to evaluate the tribocorrosion resistance of Inconel 718 in an alkaline environment consisting of a borate solution containing 0.3 M H_3_BO_3_ and 0.075M Na_2_B_4_O_7_·10H_2_O at pH 8.2. The investigation was performed on samples manufactured by conventional casting and by additive manufacturing using the PBF-LB/M method.

To accomplish this objective, the following research tasks were established:

• Comparison of the tribocorrosion resistance of samples produced by two different manufacturing methods in a borate solution containing 0.3 M H_3_BO_3_ and 0.075 M Na_2_B_4_O_7_·10H_2_O at pH 8.2;
• Determination of the effect of heat treatment on the microstructure, microhardness, and tribocorrosion resistance of both conventionally cast and additively manufactured materials;
• Investigation of the wear process, including the evolution of the coefficient of friction and electrochemical current, as well as identification of the mechanisms responsible for the degradation of IN718 samples subjected to counter-sample interaction during ball-on-plate testing.

The conducted investigations and the obtained results contributed to filling the existing knowledge gap regarding the tribocorrosion resistance of IN718 produced using different manufacturing methods in an alkaline environment. The experiments were performed in an alkaline borate solution because borate buffer electrolytes are used as model environments for investigating passivation phenomena and passive film stability on corrosion-resistant alloys, including Ni- and Ni-Cr-based materials. Their buffering capacity provides stable electrochemical conditions and facilitates the formation and investigation of passive surface films under controlled conditions [38,39,40]. Since tribocorrosion of passive materials is strongly influenced by depassivation–repassivation processes occurring during sliding, an electrolyte promoting stable passivation was selected for the present study. In addition, many industrial tribological systems, such as pumps, valves, hydraulic components, cooling circuits, and equipment operating in metalworking fluids, are exposed to alkaline environments. Consequently, the selected alkaline borate solution provides a relevant model environment for investigating tribocorrosion mechanisms under conditions representative of a wide range of engineering applications, while ensuring stable electrochemical conditions [41,42]. The selected electrolyte was also used to study degradation mechanisms of metallic materials under simultaneous mechanical and electrochemical loading in the author’s previous research [41].

## 2. Materials and Methods

**Figure 1:**
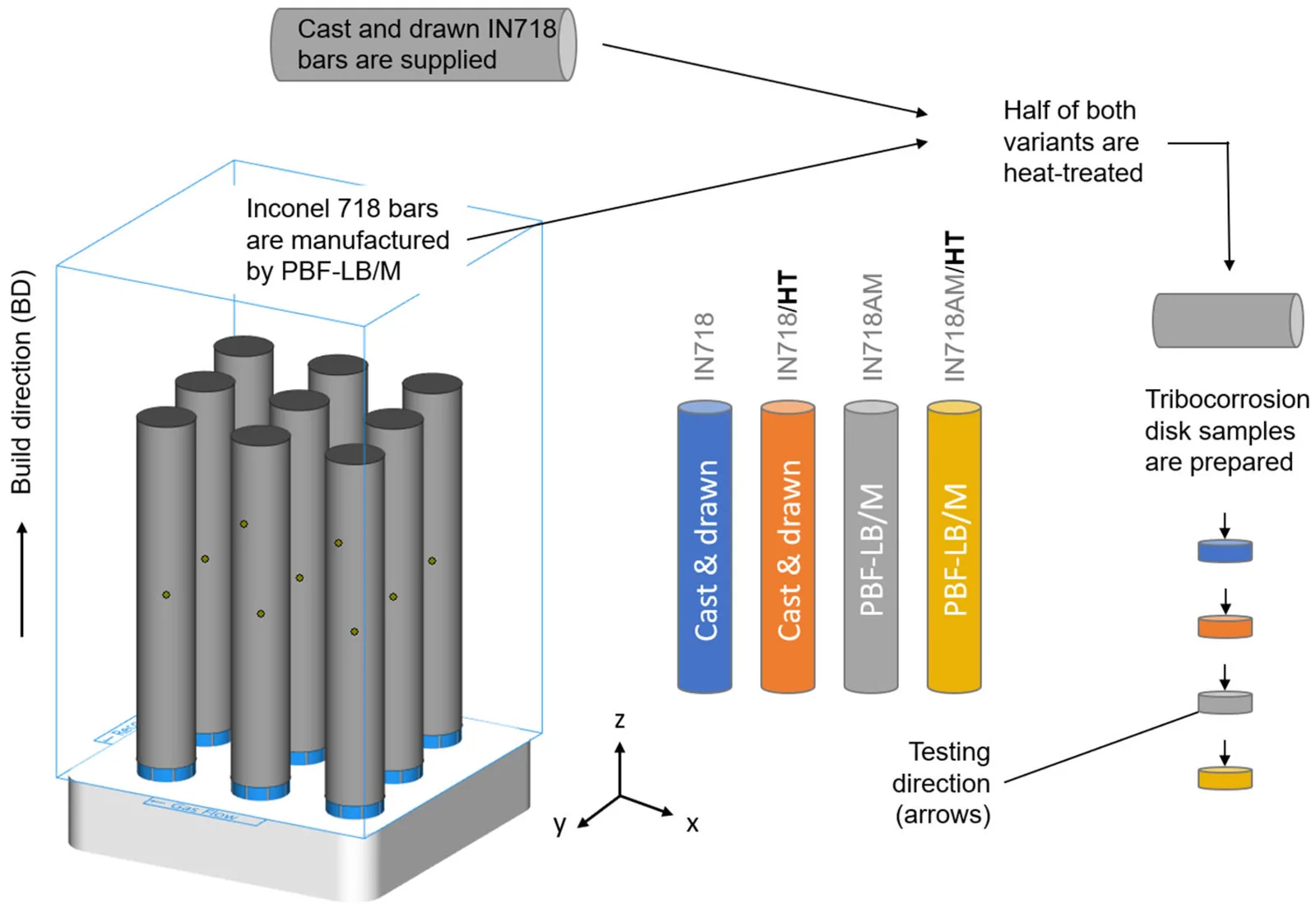
Schematic illustration of the fabrication and sample preparation process.

Samples were manufactured by additive manufacturing using an SLM 280 Dual 2.0 PBF-LB/M system (SLM Solutions Group AG, Lübeck, Germany). The system was equipped with a primary laser source with a maximum power of 700 W and a laser spot diameter of 100 µm. The samples were fabricated under a protective atmosphere of technical-grade argon with a purity class of 4.6. A 67° rotated stripe scanning strategy with a stripe width of 7 mm was applied using the following key process parameters: laser power of 300 W, scanning speed of 1300 mm/s, layer thickness of 30 µm, and a hatch distance of 0.12 mm. In a single PBF-LB/M process, cylindrical semi-finished samples with a diameter of 16 mm and a height of 100 mm were produced and oriented vertically with respect to the build direction, as shown in Figure 1. The cylindrical samples were fabricated on an Inconel 625 platform measuring 98 × 98 × 15 mm, which was preheated to 200 °C before the process and maintained at this temperature throughout fabrication.

After completion of the PBF-LB/M process, the samples and the build platform were cooled inside the machine to room temperature without any additional heat treatment. Inconel 718 (IN718) powder was supplied by the PBF-LB/M system manufacturer (SLM Solutions Group AG, Lübeck, Germany). The powder consisted of spherical particles with a nominal particle size distribution ranging from 15 to 45 µm and a chemical composition according to the manufacturer’s certificate, as presented in Table 1. The powder was provided with a certificate of compliance according to EN 10204:2004 [43] issued by the manufacturer. The alloy manufacturing protocol was described previously in [11].

Reference samples were produced using conventional manufacturing methods involving casting followed by drawing to a diameter of 10 mm. The rods were manufactured by Carpenter Technology in the annealed condition, with a diameter of 10 mm and a length of 0.5 m. The annealing treatment was performed by the manufacturer at 955 °C. No further heat treatment, including aging, was applied during the manufacturing process. The chemical composition of the rod material, compliant with the ASTM B637 standard [44], is presented in Table 2.

Samples produced using the two manufacturing methods without any additional post-processing were investigated. The samples were designated as follows:

• IN718—conventionally manufactured (cast and drawn) Inconel 718 without additional heat treatment;
• IN718AM—Inconel 718 manufactured by the PBF-LB/M process without additional heat treatment.

In addition, both conventionally manufactured and additively produced samples were subjected to heat treatment according to the following procedure:

• Stage 1: stress-relief treatment:
  ○ IN718AM: at 1065 °C for 1 h followed by furnace cooling;
  ○ IN718: at 955 °C for 1 h followed by air cooling;
• Stage 2: solution treating at 1100 °C for 1 h followed by air quenching (IN718, IN718AM);
• Stage 3: aging at 720 °C for 8 h, followed by cooling to 620 °C and holding for 8 h, and finally air cooling (IN718, IN718AM).

The samples subjected to the above heat treatment procedure were designated as follows:

• IN718HT—conventionally manufactured (cast and drawn) Inconel 718 after heat treatment;
• IN718AM/HT—PBF-LB/M manufactured Inconel 718 after heat treatment.

Microstructural observations were performed using a MIRA-3 scanning electron microscope (TESCAN, Brno, Czech Republic) on cross-sections of the samples. Both secondary electron (SE) and backscattered electron (BSE) imaging modes were employed at various magnifications. Prior to examination, all samples were ground using SiC abrasive papers with grit sizes ranging from 80 to 2000, polished with an alumina suspension, and subsequently etched in Kalling’s reagent for 10–20 s. During the microscopic examination, the chemical composition of the samples was analyzed by energy-dispersive X-ray spectroscopy (EDS) using an Ultim Max spectrometer (Oxford Instruments, High Wycombe, UK). The elemental composition was evaluated using Aztec Energy Live Standard software. Point analysis was performed to determine the chemical composition of selected areas, while EDS mapping was employed to identify and assess chemical segregation within the material.

Microhardness measurements were performed on cross-sections of the samples. For each material, 15 measurements were taken, and the results, together with the arithmetic mean values, are presented in the next section of the paper. The measurements were conducted using an FM-810 microhardness tester (Future-Tech, Kawasaki, Japan) equipped with FT-Zero automatic indentation measurement software. The microhardness tests were performed under a load of 50 g with a dwell time of 15 s.

To investigate the tribocorrosion behavior of Inconel 718 produced using two different manufacturing methods, a borate solution containing 0.3 M H_3_BO_3_ and 0.075 M Na_2_B_4_O_7_·10H_2_O at pH 8.2 was used as the test environment. The alkaline solution was purchased from Sigma-Aldrich (Burlington, MA, USA). Tribocorrosion tests were performed in the borate solution using a ball-on-plate tribometer operating in a linear reciprocating sliding mode and coupled with a potentiostat and a three-electrode electrochemical cell. An alumina (Al_2_O_3_) ball served as the counter sample and slid against the Inconel 718 sample, which acted as the working electrode. A Hg/Hg_2_SO_4_ (MSE) electrode was used as the reference. The normal load was set to 2.7 N. The reciprocating motion of the alumina ball was characterized by a frequency of 1 Hz, a sliding speed of 20 mm/s, and a dwell time of 0.25 s at the end of each stroke. The stroke length was 5 mm, and a total of 1800 reciprocating cycles were performed during each test. The work electrode is tested sample, platiunum wire is counter electrode and MSE electrode was the reference one. The normal load was set to 2.7 N. The reciprocating motion of the alumina ball was characterized by a frequency of 1 Hz, a sliding speed of 20 mm/s, and a dwell time of 0.25 s at the end of each stroke. The tests were performed in the XY plane of each sample (Figure 1).

Tribocorrosion tests were conducted under potentiostatic conditions using different applied potentials according to the following sequence:

• Measurement of the open-circuit potential (OCP) for 5 min;
• Current monitoring for 20 min at a constant potential within the passive region (−0.5 V vs. MSE) and the cathodic region (−0.9 V vs. MSE) of the alloy;
• Simultaneous measurement of current and friction forces during sliding for 30 min while maintaining the applied potential;
• Stop the sliding, followed by current monitoring for an additional 5 min at the same potential.

Wear tests performed under polarization at a potential within the passive region were used to determine the tribocorrosion wear (W_T_). When analyzing tribocorrosion wear, it is common practice to determine the component of material loss caused exclusively by mechanical (friction-induced) interactions, noted here as (W_M_). One method of determining this component (the mechanical component of tribocorrosion) is to perform wear tests in the same environment as the tribocorrosion experiments, but under cathodic polarization conditions. The presence of the corrosive medium ensures identical tribological contact conditions, while cathodic polarization suppresses corrosion processes. In the present study, wear tests performed under cathodic polarization at −0.9 V vs. MSE were used to determine the value of the mechanical wear component (W_M_). All tribocorrosion tests were conducted at room temperature (~20 °C) and were repeated three times for each sample and testing condition.

The damage generated during the tests, as well as the morphology of the wear tracks and the wear mechanisms occurring on the sample surface, were evaluated by analyzing the wear tracks using a scanning electron microscope (ZEISS EVO 10, Carl Zeiss GmbH, Oberkochen, Germany).

Volumetric wear under tribocorrosion conditions (W_T_) and under cathodic polarization (W_M_) was determined based on profilometric measurements of the wear tracks (optical profilometry, Profilm 3D, Filmetrics, San Diego, CA, USA). The wear track profiles were recorded at three locations along the length of each wear track. For each profile, the cross-sectional area of the wear track was calculated. Subsequently, the mean cross-sectional area obtained from the three measurements was multiplied by the wear-track length (5 mm). In this way, the volumetric wear was determined. The wear track profiles were recorded at three locations along the length of each wear track. For each profile, the cross-sectional area of the wear track was calculated. Subsequently, the mean cross-sectional area obtained from the three measurements was multiplied by the wear-track length (5 mm). In this way, the volumetric wear was determined.

## 3. Results

**Figure 2:**
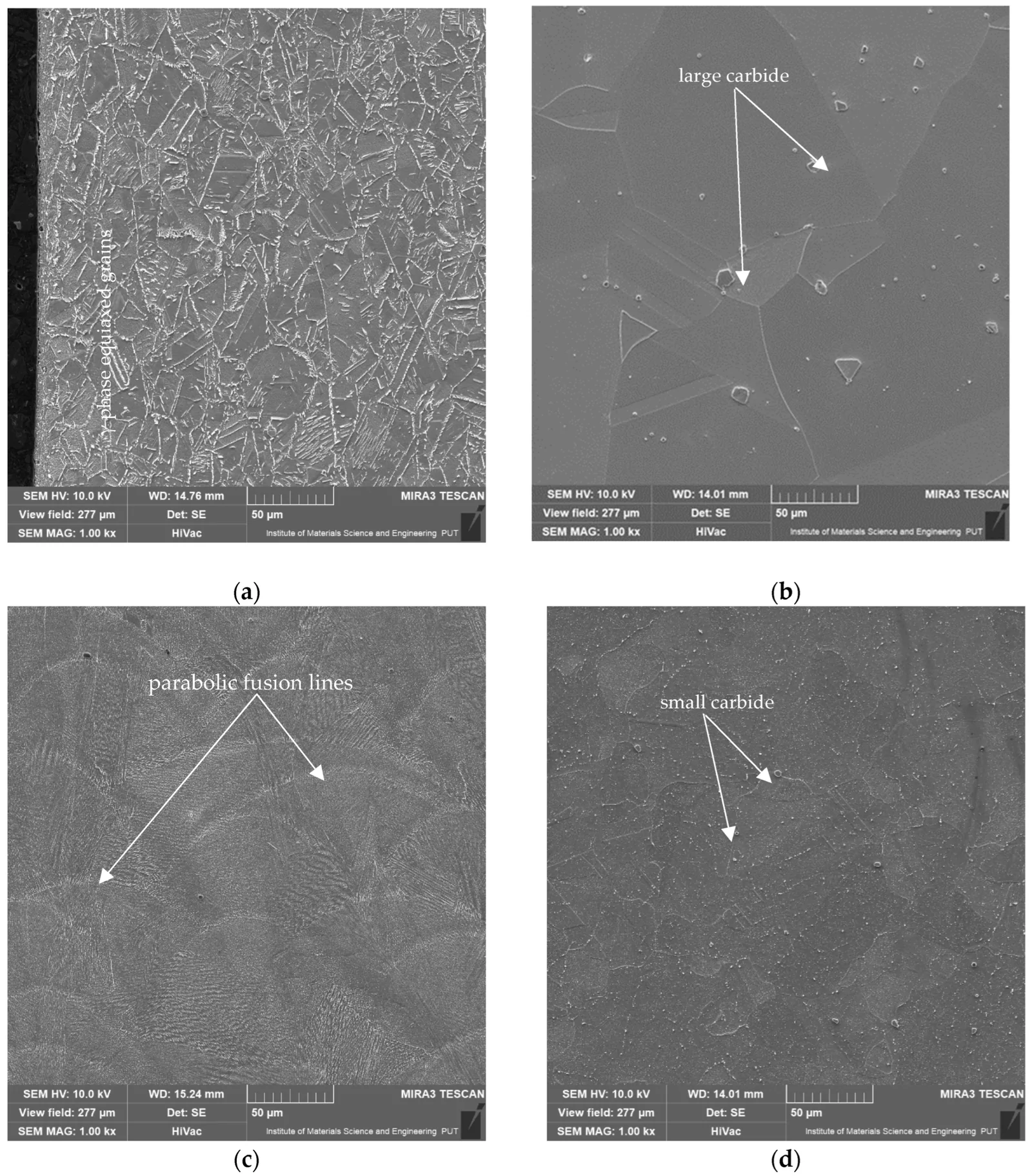
Microstructure of the tested materials on longitudinal cross-sections, i.e., parallel to the drawing axis of the conventionally manufactured rods and to the build direction of the PBF-LB/M samples: (**a**) IN718, (**b**) IN718HT, (**c**) IN718AM, (**d**) IN718AM/HT.

Microstructural characterization was first performed for both conventionally manufactured and additively produced samples. The analysis also included samples subjected to heat treatment. Representative microstructures of all investigated materials are presented in Figure 2. The microstructure of Inconel 718 alloy is composed of a supersaturated solid solution rich in nickel, chromium and iron (γ phase equiaxed grains) with precipitates of phases enriched in niobium compounds. Previous studies [11,45] show that the characteristic precipitates are carbides enriched in niobium and titanium. In contrast, the matrix is predominantly composed of Ni, Cr, and Fe [11,45].

The microstructure of conventionally manufactured Inconel 718 (IN718 and IN718HT) exhibited a larger grain size than that of the additively manufactured material (IN718AM and IN718AM/HT). As a result of the high temperatures applied during the SR, SA, and AA treatments, the additively manufactured samples no longer exhibited the characteristic PBF-LB/M microstructure, which was described in detail in our previous study [11]. Instead, they consisted of semi-equiaxed γ grains containing numerous twin boundaries. The grain size of the as-built IN718AM material reported in the previous study [11] was comparable to that observed in the as-received conventionally manufactured alloy. However, heat treatment resulted in a significant increase in grain size of the conventionally manufactured alloy. As reported in the literature [46,47,48], PBF-LB/M manufactured Inconel 718 exhibits high resistance to grain growth, even after exposure to temperatures of up to 1180 °C for holding times of up to 12 h. The size and distribution of precipitates observed within the grains depended on the applied heat treatment method. Qualitative analysis indicated that these precipitates most likely consisted of niobium- and titanium-rich carbides. Their presence is expected because the initial microsegregation occurring in PBF-LB/M processed Inconel 718 promotes the formation of (Nb,Ti)C carbides during the early stages of high-temperature homogenization [48]. These carbides are beneficial, as they restrict grain growth through the Zener pinning effect. Importantly, the microstructural analysis of the additively manufactured samples did not reveal the characteristic PBF-LB/M defects, such as lack-of-fusion (LOF) pores, in the examined cross-sections.

The Inconel 718 material produced by the PBF-LB/M method has a fine-grained microstructure with characteristic parabolic fusion lines and columnar γ grains. The grains are elongated in the build direction, following the high temperature gradient during the process. That alloy is characterized by homogeneity of the chemical composition, which was confirmed in our previous publication [11]. In the case of the Inconel 718 alloy sample produced by the PBF-LB/M method, the presence of carbides was found to be lower than in the case of production by the conventional method.

The microhardness results obtained for the investigated materials are summarized in Table 3. The highest hardness was recorded for the heat-treated additively manufactured samples (IN718AM/HT), which exhibited an average hardness of 522 HV. In contrast, the lowest average hardness was observed for the conventionally manufactured samples without additional heat treatment (IN718). The results indicate that heat treatment increased the hardness of Inconel 718, regardless of the manufacturing method. Furthermore, the additively manufactured samples exhibited higher hardness values than conventionally produced samples. Since both variants were subjected to the same ageing treatment, the higher hardness of IN718AM/HT is more likely governed by the size and volume fraction of the strengthening γ″ and γ′ precipitates than by grain size alone. Residual Nb microsegregation inherited from the PBF-LB/M solidification structure modifies the local Nb availability during ageing and hence the resulting precipitate distribution, which has been reported to cause differences in the post-heat-treatment properties of additively manufactured and wrought IN718 [26,46]. Additionally, the microhardness values reported in Table 3 were measured under a low test force (HV0.05) and are therefore affected by the indentation size effect (ISE), which yields apparent hardness values higher than those obtained at macro-loads. For the PBF-LB/M material, the present values exceed the ranges reported for the as-built (300–350 HV) and heat-treated (420–480 HV) conditions [3] by a nearly constant margin of about 20%, which indicates a systematic load-related offset rather than a difference in material condition. The values in Table 3 should therefore be interpreted as a relative comparison between the investigated variants.

**Figure 3:**
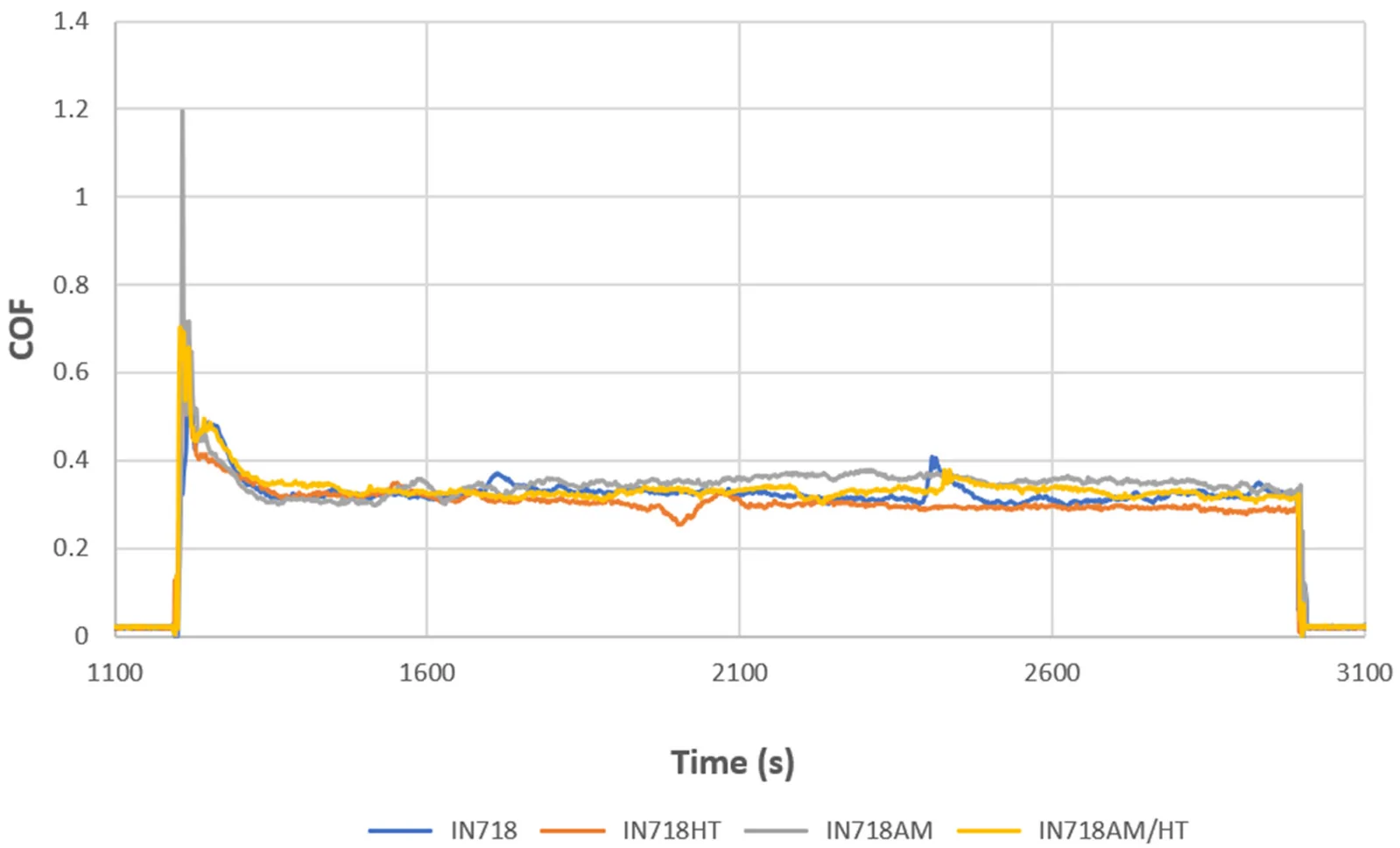
Evolution of the coefficient of friction under mechanically dominated wear conditions: IN718, IN718HT, IN718AM, and IN718AM/HT.

**Figure 4:**
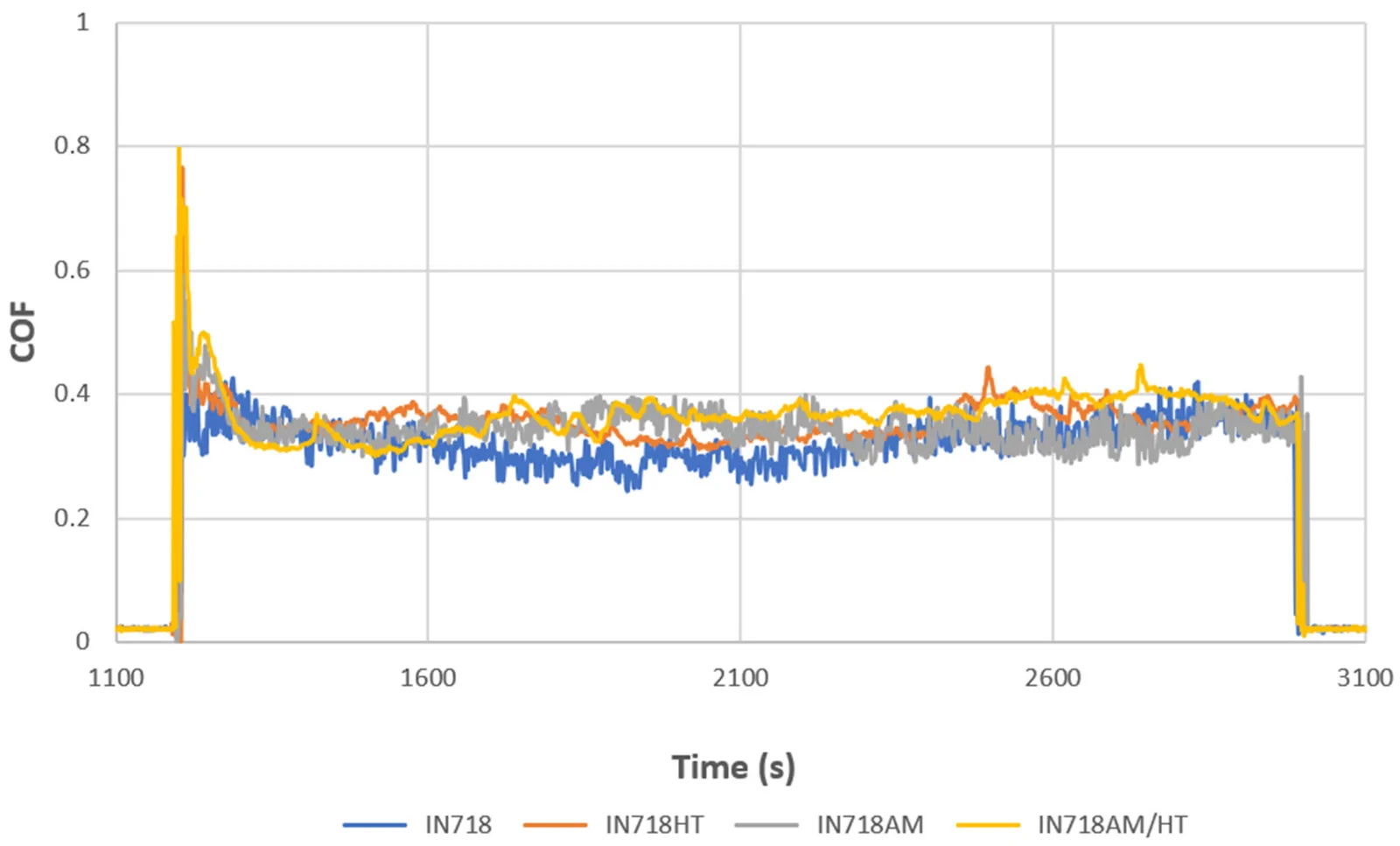
Evolution of the coefficient of friction under tribocorrosion conditions: IN718, IN718HT, IN718AM, and IN718AM/HT.

The changes in the coefficient of friction (COF) recorded during the mechanical wear and tribocorrosion tests are presented in Figure 3 and Figure 4, respectively. Under mechanically dominated wear conditions (Figure 3), the coefficient of friction remained relatively stable throughout the test for all materials investigated, despite the occurrence of short-term fluctuations. These fluctuations can be attributed to periodic formation, accumulation, and removal of wear debris within the contact zone. No substantial differences in the average coefficient of friction were observed between the conventionally manufactured and PBF-LB/M produced materials. Similarly, the applied heat treatment did not affect the friction behavior of the alloys investigated. A comparable trend was observed under tribocorrosion conditions (Figure 4). The friction curves exhibited fluctuations of similar magnitude to those recorded during mechanically dominated wear. The similarity of the friction curves obtained for all material variants suggests that the friction behavior of Inconel 718 in the investigated environment was largely independent of the manufacturing method and post-processing condition. In contrast, the wear results presented in Table 4 revealed measurable differences in material loss between the materials investigated. Consequently, the observed variations in tribocorrosion performance should be attributed primarily to differences in wear resistance rather than to changes in friction behavior. The improved wear resistance observed after heat treatment is therefore more likely associated with the increase in material hardness (Table 3) than with any significant modification of the coefficient of friction.

**Figure 5:**
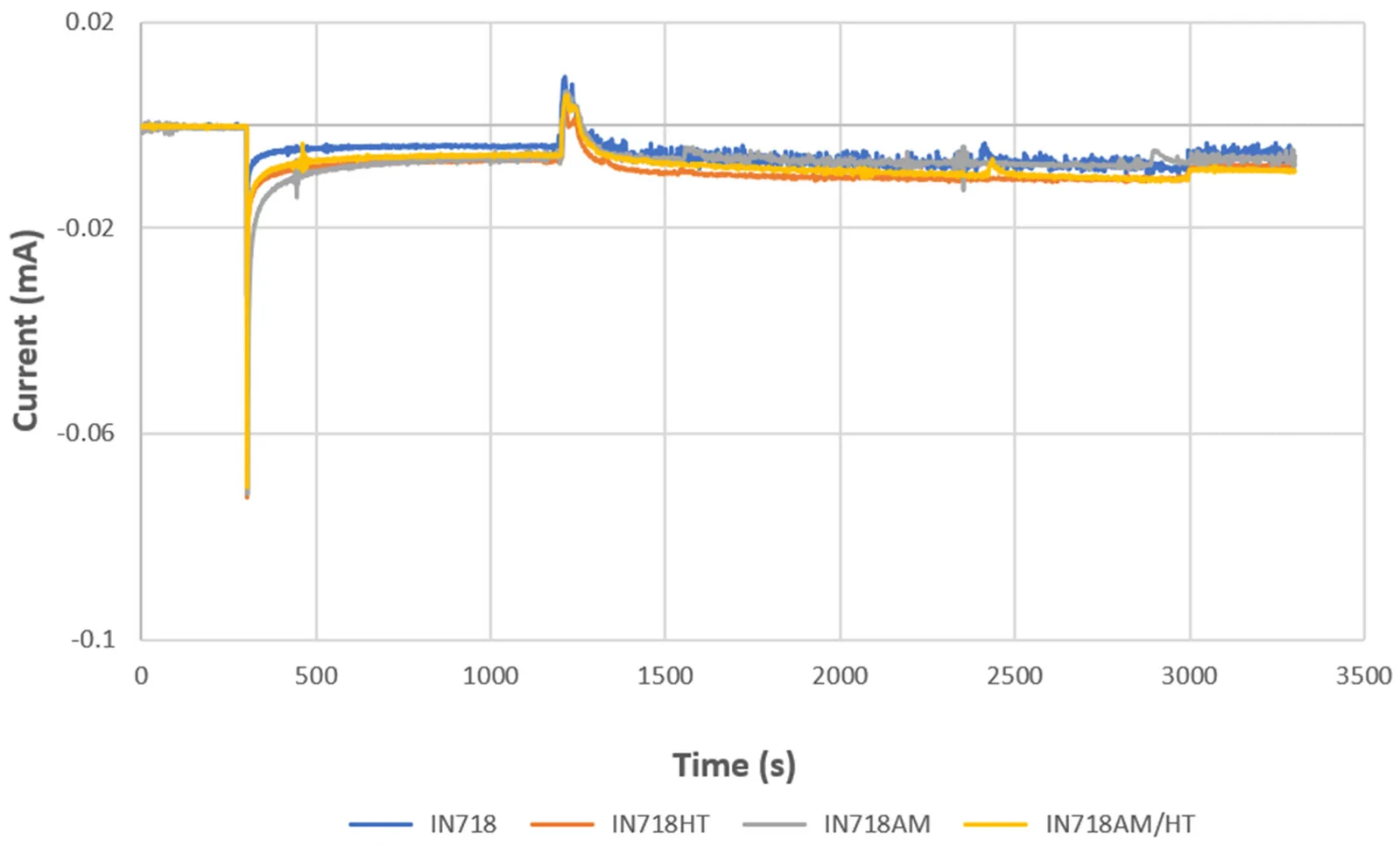
Evolution of the current under mechanically dominated wear tests: IN718, IN718HT, IN718AM, and IN718AM/HT.

**Figure 6:**
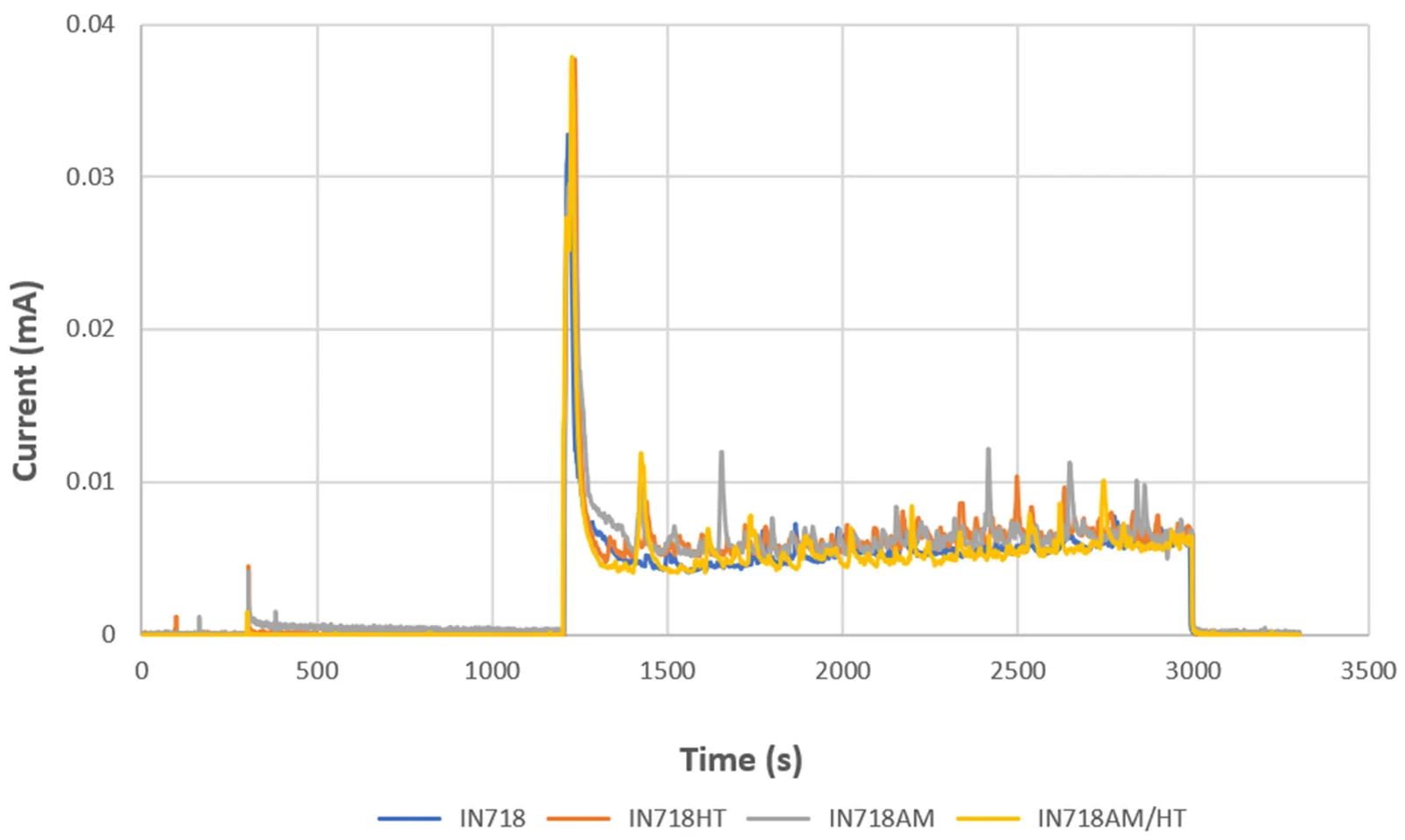
Evolution of the current under tribocorrosion tests: IN718, IN718HT, IN718AM, and IN718AM/HT.

The changes in the current measured during the mechanical wear and tribocorrosion tests are presented in Figure 5 and Figure 6, respectively. Under mechanically dominated wear conditions (Figure 5), the measured current remained relatively low and stable throughout the test. No significant differences were observed between the materials investigated. In contrast, the current transients recorded during the tribocorrosion tests (Figure 6) are characteristic of passive alloys subjected to simultaneous mechanical and electrochemical loading. In all cases, the current decreased during the initial immersion period owing to the formation and growth of a passive oxide film on the alloy surface. Upon the initiation of sliding, the current increased sharply and remained at an elevated level throughout the test. This behavior resulted from mechanical removal of the passive film by the counter sample and the consequent exposure of bare metal to the corrosive environment, which intensified electrochemical reactions at the contact interface. Following the cessation of sliding, the current rapidly decreased towards its initial value, reflecting reformation of the passive oxide layer on the exposed surface. Overall, the similar steady-state current levels observed for all investigated variants suggest comparable electrochemical behavior under tribocorrosion conditions, despite differences in manufacturing methods and heat treatment state.

The volumetric wear results obtained under different testing conditions, namely tribocorrosion (W_T_) and mechanically dominated wear (W_M_), are summarized in Table 4. The extreme measurement results and the mean value with standard deviation are provided. Specific wear rates were also calculated. Under mechanically dominated wear loading conditions, the lowest average volumetric wear was recorded for the heat-treated PBF-LB/M manufactured samples (IN718AM/HT), amounting to 0.549 ± 0.081 × 10^−3^ mm^3^. In contrast, the highest material loss, equal to 0.747 ± 0.092× 10^−3^ mm^3^, was observed for the conventionally manufactured samples without heat treatment (IN718). The IN718 samples also exhibited the highest volumetric wear following the tribocorrosion tests. The measured material loss reached 0.906 ± 0.099 × 10^−3^ mm^3^. The lowest material loss under tribocorrosion conditions was observed for the heat-treated additively manufactured samples (IN718AM/HT), for which the wear volume amounted to 0.719 ± 0.096 × 10^−3^ mm^3^.

The additively manufactured samples exhibited a lower average wear volume under the investigated conditions. In all cases, the tribocorrosion tests resulted in higher material loss than the corresponding mechanically dominated wear tests, highlighting the detrimental effect of the synergistic interaction between wear and corrosion on material degradation.

**Figure 7:**
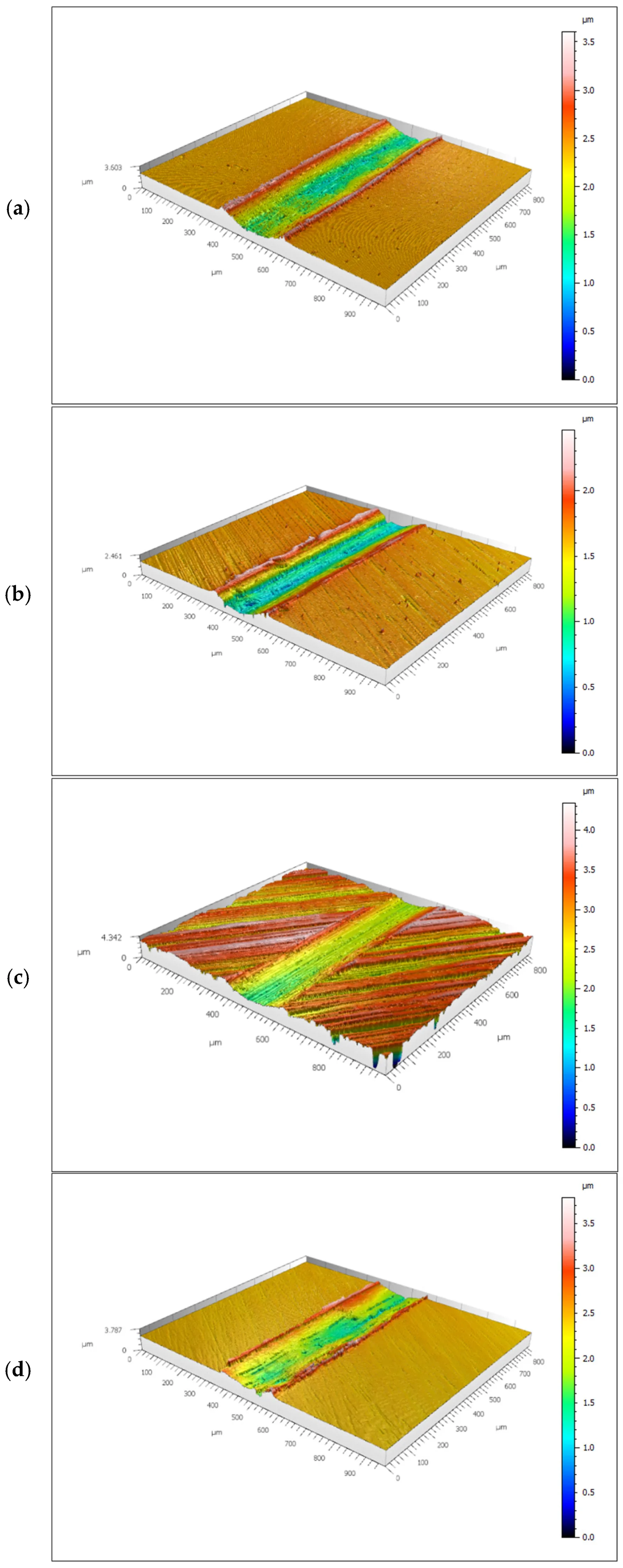
Wear track profile under mechanically dominated wear conditions: (**a**) IN718, (**b**) IN718HT, (**c**) IN718AM, (**d**) IN718AM/HT.

**Figure 8:**
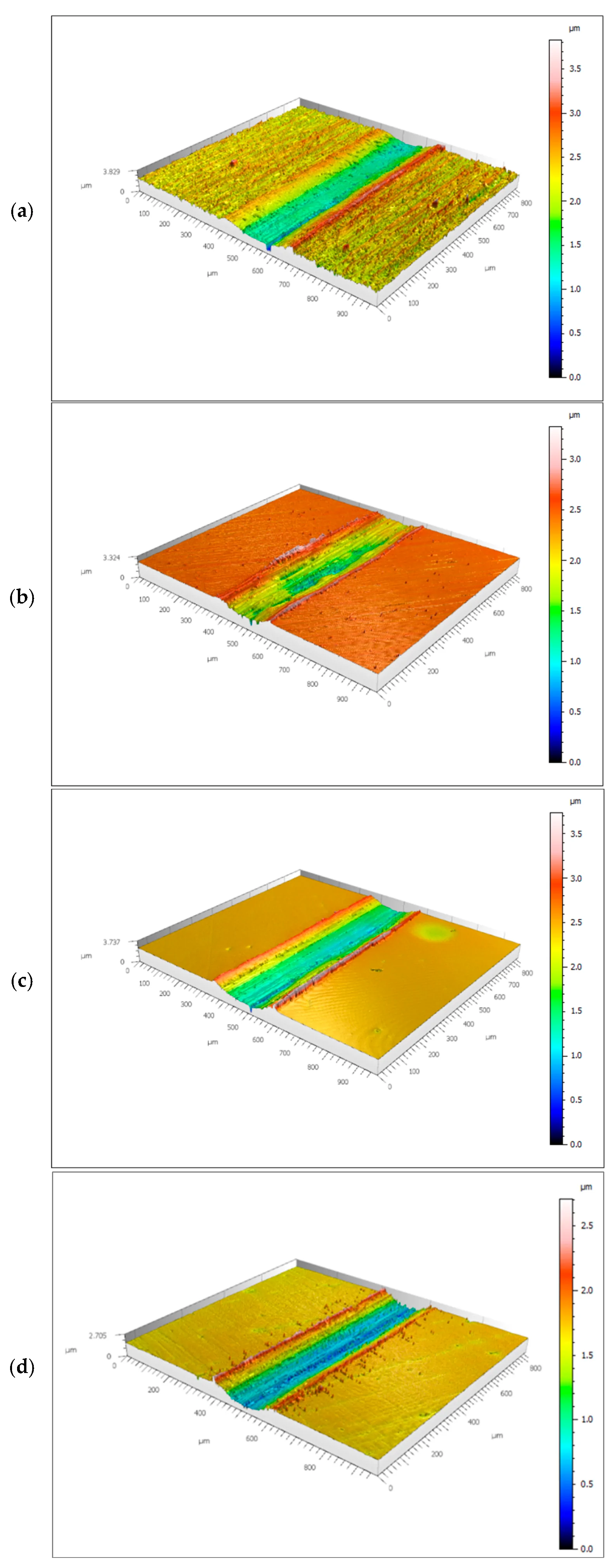
Wear track profile under tribocorrosion conditions: (**a**) IN718, (**b**) IN718HT, (**c**) IN718AM, (**d**) IN718AM/HT.

Figure 7 and Figure 8 present representative wear track profiles obtained by optical profilometry after the mechanical wear and tribocorrosion tests. The profiles illustrate the material loss caused by the interaction between the counter sample and the sample surface. Under mechanically dominated wear conditions (Figure 7), the deepest wear tracks were observed for the conventionally manufactured material without heat treatment (IN718), whereas the shallowest profiles corresponded to the heat-treated PBF-LB/M produced material (IN718AM/HT). A similar trend was observed under tribocorrosion conditions (Figure 8). Although all wear tracks were deeper than those produced under mechanically dominated wear loading, the relative ranking of the investigated materials remained unchanged.

**Figure 9:**
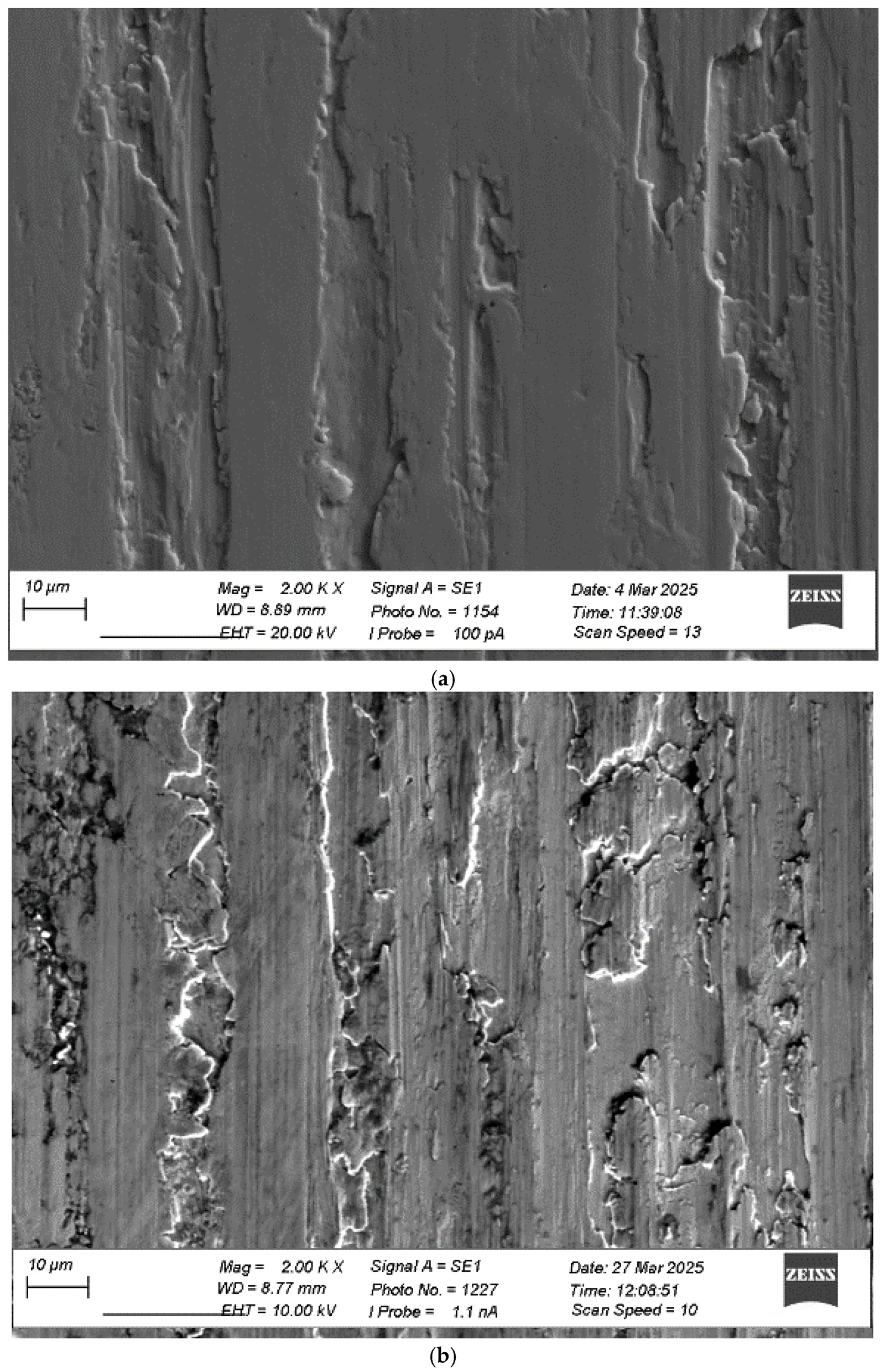

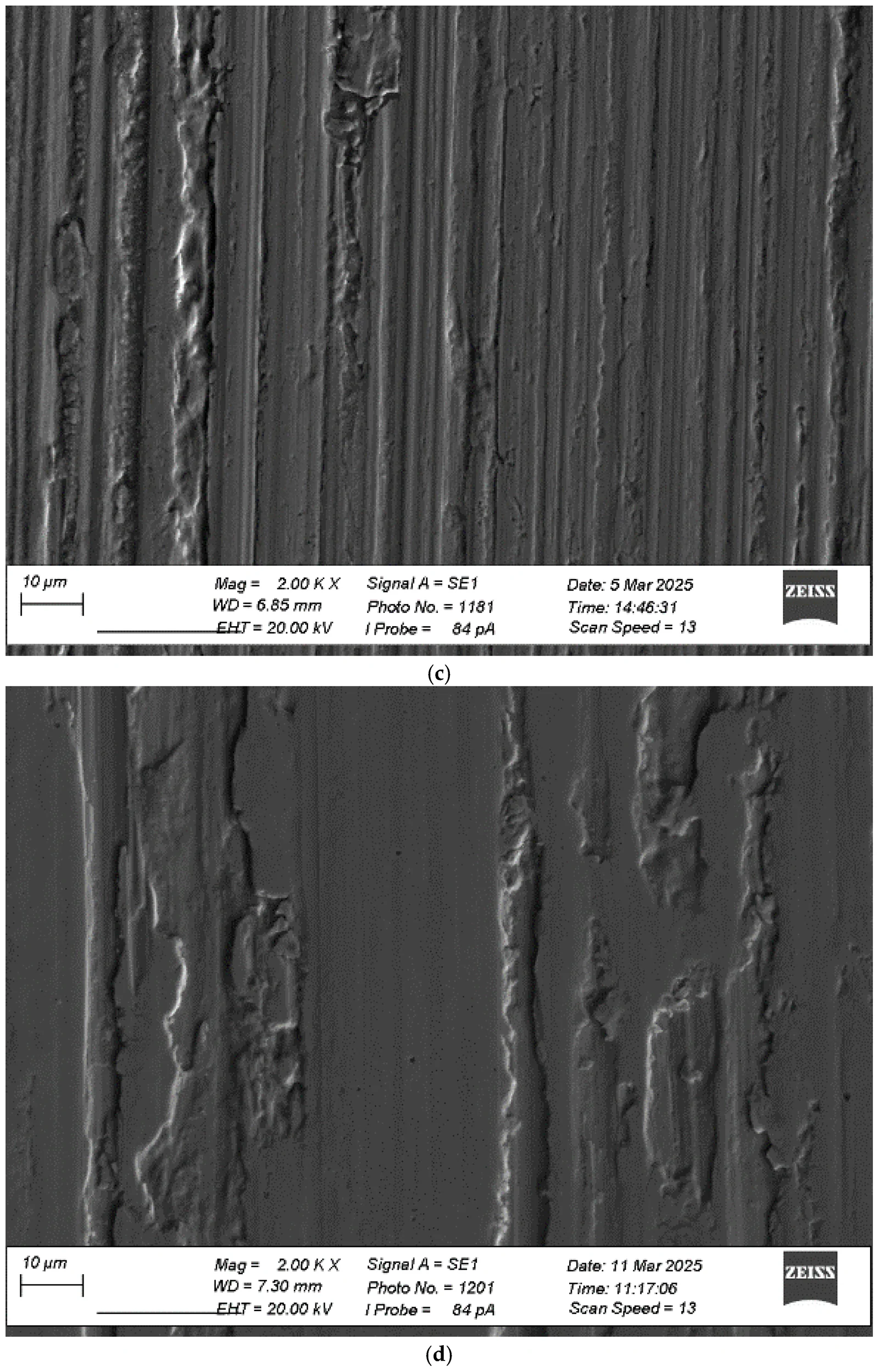
Wear track surface samples under mechanically dominated wear conditions: (**a**) IN718, (**b**) IN718HT, (**c**) IN718AM, (**d**) IN718AM/HT.

**Figure 10:**
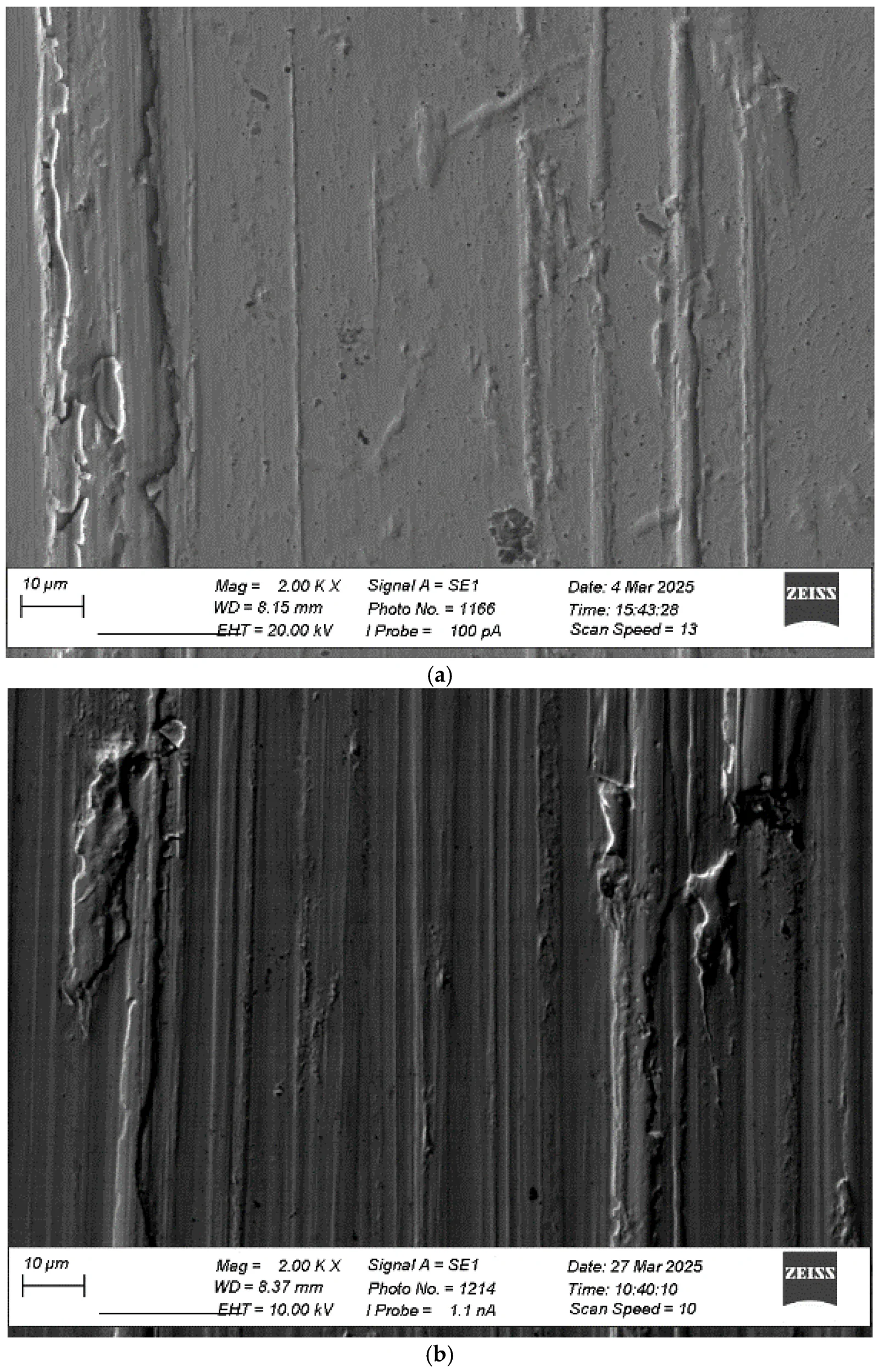

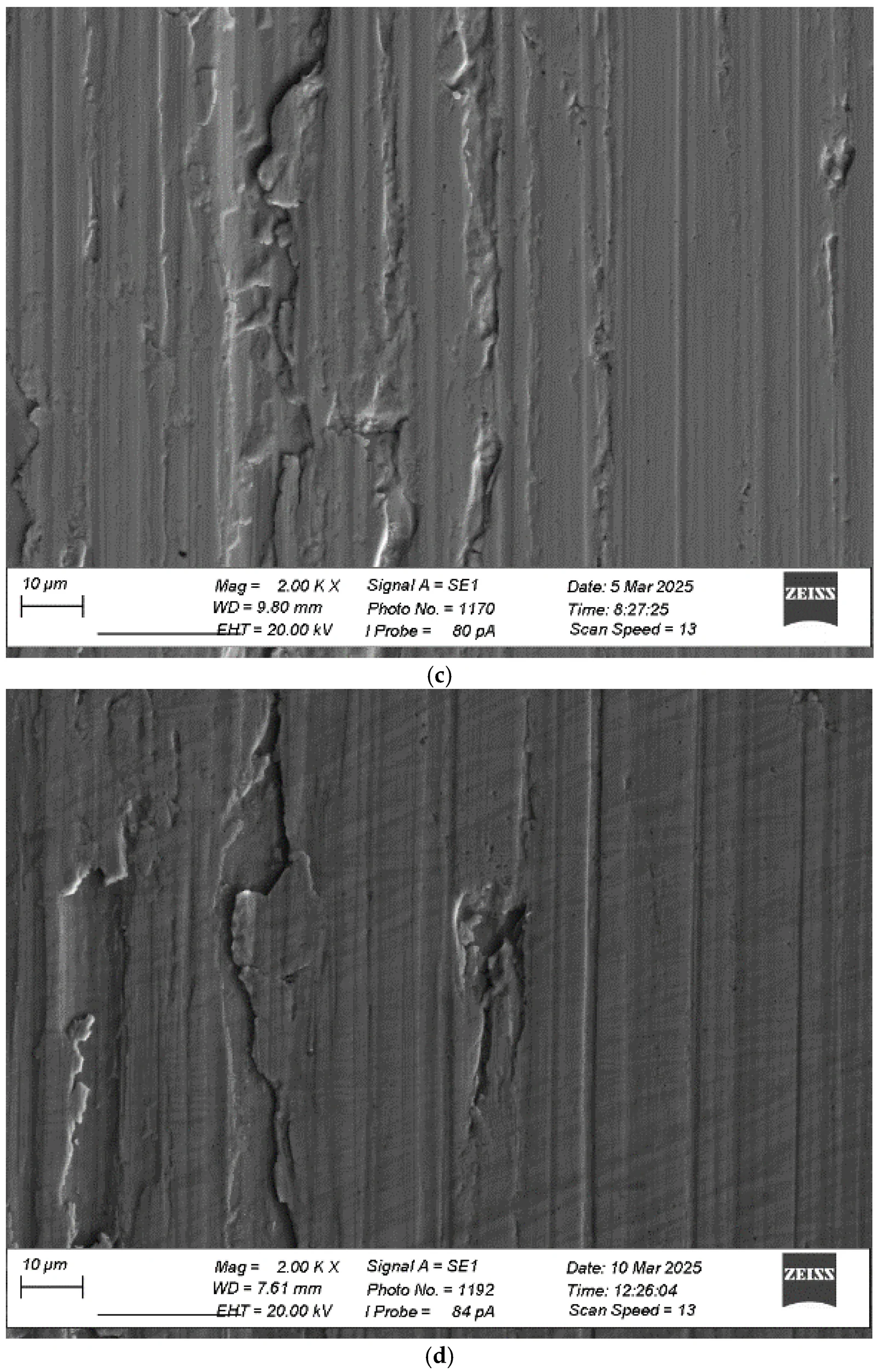
Wear track surface samples under tribocorrosion conditions: (**a**) IN718, (**b**) IN718HT, (**c**) IN718AM, (**d**) IN718AM/HT.

Figure 9 and Figure 10 present SEM micrographs of the wear track surfaces. For all test variants, including both mechanically dominated wear and tribocorrosion conditions, features characteristic of abrasive wear were observed. These features consisted primarily of parallel grooves formed as a result of micro-scratching of the sample surface by the alumina counter sample during sliding. In the wear tracks generated under tribocorrosion conditions, these grooves were less pronounced and less numerous than those observed after mechanically dominated wear tests. This observation suggests that part of the surface damage produced by the mechanical interaction between the counter sample and the sample could be removed or altered by the simultaneous action of corrosion processes occurring during tribocorrosion.

For the tribocorrosion wear (W_T_) values presented in Table 4, the following analyses were performed:

• Evaluation of the statistical significance of differences in the mean volumetric wear using a two-tailed Student’s *t*-test;
• Assessment of the practical significance of the observed differences between the mean volumetric wear values using Cohen’s effect size coefficient (Cohen’s d).

The results of these analyses are summarized in Table 5.

## 4. Discussion

Figure 6 shows the current transients recorded during the tribocorrosion tests. The obtained results do not indicate significant differences in the corrosion behavior of the investigated IN718 variants. The current transients are characteristic of passive materials subjected to tribocorrosion. In all cases, the current decreased during the initial immersion period owing to the formation and growth of a passive oxide film on the alloy surface, which reduced the anodic dissolution rate of the exposed metal [49,50]. Upon the initiation of sliding, the current increased sharply and remained at an elevated level throughout the test. This behavior reflected the mechanical disruption of the passive film by the counter sample and the exposure of bare metal to the corrosive environment. Similar current transients were widely reported for passive alloys subjected to tribocorrosion, where sliding continuously removes the passive film, promotes depassivation, and accelerates electrochemical dissolution until repassivation occurs [49,50,51]. As a consequence, the depassivation of the surface and the continuous removal of corrosion products during sliding increased the electrochemical activity of the system, resulting in higher current values. The proposed depassivation–repassivation mechanism is an interpretation based on the electrochemical response.

The current stabilized at approximately 0.006–0.008 mA for all investigated materials during sliding. The steady-state current observed during this stage can be attributed to a dynamic balance between mechanically induced depassivation and electrochemical repassivation processes occurring within the contact zone [50,51]. However, pronounced transient current peaks were observed immediately after the initiation of sliding. The highest peaks, reaching approximately 0.035–0.040 mA, were recorded for the non-heat-treated materials (IN718 and IN718AM), whereas the heat-treated samples (IN718HT and IN718AM/HT) exhibited substantially lower peak values, generally below 0.015 mA. After the termination of sliding, the current rapidly decreased, reflecting the reformation of the passive oxide film on the exposed surface. Similar repassivation behavior was reported for numerous passive alloys under tribocorrosion conditions, where the cessation of mechanical action enables restoration of the protective oxide layer and a corresponding reduction in the corrosion rate [49,50]. The similar steady-state current levels observed for all investigated material variants suggest comparable electrochemical behavior and passivation characteristics under tribocorrosion conditions, despite differences in manufacturing methods and heat-treatment state.

The wear track surface morphologies shown in Figure 9 indicate that abrasive wear was the dominant material removal mechanism under both mechanically dominated wear and tribocorrosion conditions. In the case of micro-cutting, according to the Archard wear model, the volumetric wear is largely governed by the hardness of the material. The microhardness results presented in Table 3 confirm this relationship. A decrease in volumetric wear was observed with increasing material hardness for all investigated samples.

The wear resistance results presented in Table 4 indicate that, for each of the four investigated IN718 variants, tribocorrosion wear was considerably higher than that observed under mechanically dominated wear loading conditions. The greater material loss recorded during tribocorrosion may be attributed to the synergistic interaction between friction and corrosion. This effect is most likely associated with the repassivation process described previously, involving the continuous removal and reformation of the passive oxide layer as a result of sliding-induced surface damage.

Under tribocorrosion conditions, lower material loss was observed for the additively manufactured samples both in the manufactured condition and after heat treatment. It should be noted, however, that the observed differences in tribocorrosion resistance were not statistically significant (Table 5). The lower average volumetric wear measured for the additively manufactured samples under tribocorrosion conditions may be attributed to their higher hardness values (Table 3).

Analysis of the material loss results presented in Table 4 for tribocorrosion wear (W_T_) and under mechanically dominated wear loading conditions (W_M_) under different manufacturing and treatment conditions indicates a beneficial effect of heat treatment (HT) on the wear and tribocorrosion resistance of Inconel 718. Under tribocorrosion conditions, the wear volume of the base material (IN718) amounted to 0.906 ± 0.099 × 10^−3^ mm^3^, whereas heat treatment (IN718HT) reduced the wear volume to 0.772 ± 0.088 × 10^−3^ mm^3^, corresponding to a reduction of approximately 15%. A similar trend was observed for the additively manufactured material. For IN718AM, the average wear volume decreased from 0.888 ± 0.095 × 10^−3^ mm^3^ to 0.719 ± 0.096 × 10^−3^ mm^3^ after heat treatment (IN718AM/HT), representing a reduction of approximately 19%.

An even greater effect of heat treatment was observed under mechanically dominated wear loading conditions. For the conventionally manufactured material (IN718), the wear volume decreased from 0.747 ± 0.092 × 10^−3^ mm^3^ to 0.579 ± 0.076 × 10^−3^ mm^3^ after heat treatment (IN718HT), corresponding to a reduction of approximately 22%. Similarly, for the additively manufactured material, the wear volume decreased from 0.717 ± 0.094 × 10^−3^ mm^3^ for IN718AM to 0.549 ± 0.081 × 10^−3^ mm^3^ for IN718AM/HT, representing a reduction of approximately 23%.

The statistical analysis based on Student’s *t*-test (Table 5) did not reveal statistically significant differences for any of the comparisons investigated. However, the effect size analysis using Cohen’s d indicated that some of the observed differences may be of practical importance. Two main conclusions can be drawn from the results presented in Table 5:

• The manufacturing method (conventional or additive) did not affect the volumetric wear under tribocorrosion conditions; this conclusion applies to both the manufactured and heat-treated materials;
• The applied heat treatment showed a tendency to reduce tribocorrosion wear, particularly in the case of the additively manufactured samples, for which the largest practical effect was observed (d = 1.10 for the comparison between IN718AM and IN718AM/HT).

Therefore, although the observed differences did not reach statistical significance at the adopted significance level, the effect size analysis suggests that heat treatment contributed to improved tribocorrosion resistance, especially for the PBF-LB/M manufactured material. This observation is consistent with the increase in hardness after heat treatment (Table 3) and the corresponding reduction in volumetric wear (Table 4).

## 5. Conclusions

Based on the results obtained in this study, the following conclusions can be drawn:

1. The applied heat treatment increased the hardness of all investigated Inconel 718 variants. Higher hardness values were observed for the additively manufactured materials (IN718AM and IN718AM/HT) compared with their conventionally manufactured samples.
2. All investigated Inconel 718 variants exhibited the ability to passivate in the borate solution containing 0.3 M H_3_BO_3_ and 0.075 M Na_2_B_4_O_7_·10H_2_O at pH 8.2.
3. For all investigated material variants, wear under tribocorrosion conditions was consistently higher than that observed under mechanically dominated wear loading conditions. This behavior may be attributed to intensified electrochemical processes initiated by friction-induced surface damage (micro-cutting) and the associated depassivation–repassivation cycles involving the continuous removal and reformation of the passive oxide film.
4. Under tribocorrosion conditions, lower material loss was observed for the additively manufactured samples both in the as-manufactured condition and after heat treatment. However, statistical analysis showed that the manufacturing method did not affect the volumetric wear under tribocorrosion conditions.
5. The results presented in Table 4 demonstrate that heat treatment reduces volumetric wear under tribocorrosion conditions for both conventionally manufactured and additively manufactured Inconel 718. The beneficial effect of heat treatment was particularly pronounced for the PBF-LB/M produced samples.

## Figures and Tables

**Table 1:** Chemical composition of the Inconel 718 powder.

Ni	Cr	Fe	Mo	Nb	Ti	Al	Ta	Co	C	Mn	Si	P
55.30	16.30	18.35	3.03	5.05	0.85	0.52	0.37	0.08	0.04	0.02	0.04	0.05

**Table 2:** Chemical composition of the Inconel 718 rod.

Ni	Cr	Fe	Mo	Nb	Ti	Al	Ta	Co	C	Mn	Si	P
53.20	18.40	17.8	2.91	5.26	0.90	0.54	0.01	0.42	0.03	0.07	0.07	0.01

**Table 3:** Average microhardness values for all samples.

HV_0.05_	IN718	IN718HT	IN718AM	IN718AM/HT
Value	326 ± 13	471 ± 17	384 ± 16	522 ± 21

**Table 4:** Results of tribocorrosion (W_T_) and mechanically dominated wear (W_M_) test.

Type of Material Production	Material loss [mm^3^] × 10^−3^				Specific Wear Rates [mm^3^/(Nm)] × 10^−5^	
	W_T_		W_M_		W_T_	W_M_
	(Min, Max)	Average	(Min, Max)	Average		
IN718	0.714–1.118	0.906 ± 0.099	0.504–0.989	0.747 ± 0.092	1.86	1.54
IN718HT	0.659–0.888	0.772 ± 0.088	0.495–0.702	0.579 ± 0.076	1.59	1.19
IN718AM	0.656–1.159	0.888 ± 0.095	0.535–0.793	0.717 ± 0.094	1.83	1.48
IN718AM/HT	0.604–0.839	0.719 ± 0.096	0.427–0.650	0.549 ± 0.081	1.48	1.13

**Table 5:** Statistical comparison of tribocorrosion wear values for the investigated materials.

Comparison	t	*p*	Cohen’s d	Conclusion
IN718-IN718/HT	1.55	0.145	0.78	No statistically significant difference; moderate-to-large effect size
IN718AM-IN718AM/HT	1.96	0.076	1.10	No statistically significant difference; large effect size (clear tendency)
IN718HT-IN718AM/HT	1.00	0.343	0.58	No statistically significant difference; moderate effect size

## Data Availability

The original contributions presented in this study are included in the article. Further inquiries can be directed to the corresponding author.

## References

[1] Zhao X., Chen J., Lin X., Huang W. (2008). Study on microstructure and mechanical properties of laser rapid forming Inconel 718. Mater. Sci. Eng. A.

[2] Iturbe A., Giraud E., Hormaetxe E., Garay A., Germain G., Ostolaza K., Arrazola P.J. (2017). Mechanical characterization and modelling of Inconel 718 material behavior for machining process assessment. Mater. Sci. Eng. A.

[3] Hosseini E., Popovich V.A. (2019). A review of mechanical properties of additively manufactured Inconel. Addit. Manuf..

[4] Zou Y., Tang S., Guo S., Song X. (2024). Tool wear analysis in turning inconel-657 using various tool materials. Mater. Manuf. Processes.

[5] Liu Q.C., Guo Q., Li C., Mei Y., Zhou X., Huang Y. (2016). Recent progress on evaluation of precipitates in Inconel 718 superalloy. Acta Metall. Sin..

[6] Zhang H., Li C., Guo Q., Ma Z., Huang Y., Li H., Liu Y. (2018). Hot tensile behavior of cold-rolled Inconel 718 alloy at 650 °C: The role of δ phase. Mater. Sci. Eng. A.

[7] Wang X., Gong X., Chou K. (2016). Review on powder-bed laser additive manufacturing of Inconel 718 parts. Proc. Inst. Mech. Eng. Part B J. Eng. Manuf..

[8] Pei C., Shi D., Yuan H., Li H. (2019). Assessment of mechanical properties and fatigue performance of a selective laser melted nickel-base superalloy Inconel 718. Mater. Sci. Eng. A.

[9] (2021). Additive Manufacturing—General Principles—Fundamentals and Vocabulary.

[10] Gruber K., Ziółkowski G., Pawlak A., Kurzynowski T. (2022). Effect of stress relief and inherent strainbased pre-deformation on the geometric accuracy of stator vanes additively manufactured from inconel 718 using laser powder bed fusion. Precis. Eng..

[11] Stachowiak A., Wieczorek D., Gruber K., Bartkowski D., Bartkowska A., Ulbrich D. (2023). Comparison of tribocorrosion resistance of Inconel^®^ 718 alloy manufactured by conventional method and laser powder bed fusion method. Tribol. Int..

[12] Stevens E.L., Toman J., To A.C., Chmielus M. (2017). Variation of hardness, microstructure, and Laves phase distribution in direct laser deposited alloy 718 cuboids. Mater. Des..

[13] Jia Q., Gu D. (2014). Selective laser melting additive manufacturing of Inconel 718 superalloy parts: Densification, microstructure and properties. J. Alloys Compd..

[14] Yan X., Gao S., Chang C., Huang J., Khanlari K., Dong D., Ma W., Fenineche N., Liao H., Liu M. (2021). Effect of building directions on the surface roughness, microstructure, and tribological properties of selective laser melted Inconel 625. J. Mater. Process. Technol..

[15] Kayali Y., Kanca E., Gunen A. (2022). Effect of boronizing on microstructure, high-temperature wear and corrosion behavior of additive manufactured Inconel 718. Mater. Charact..

[16] Doleker K.M., Erdogan A., Yener T., Karaoglanli A.C., Uzun O., Gok M.S., Zeytin S. (2021). Enhancing the wear and oxidation behaviors of the Inconel 718 by low temperature aluminizing. Surf. Coat. Technol..

[17] Tripathy M., Gaskell K., Laureto J., Davami K., Behesti A. (2023). Elevated temperature fretting wear study of additively manufactured inconel 625 superalloy. Addit. Manuf..

[18] Hatami O., Adibi H., Rezaei S.M. (2022). Application of a compressed air jet for cleaning of wheel surface in grinding nickel-based super alloy Inconel 718. CIRP J. Manuf. Sci. Technol..

[19] Ho K.C., Xu P.L., Sheu H.H., Lee H.B., Li W.K. (2025). Corrosion and Wear Behavior of Selective Laser Melting Inconel 718 Alloy After Solution Treatment. Mater. Corros..

[20] Dubey D., Mukherjee R., Singh M.K. (2024). A review on tribological behavior of nickel-based Inconel superalloy. Proc. Inst. Mech. Eng. Part J J. Eng. Tribol..

[21] Chen M., Shi X.H., Yang H., Liaw P.K., Cao M.C., Hawk J.A., Qiao J. (2018). Wear behavior of Al_0.6_CoCrFeNi high-entropy alloys: Effect of environments. J. Mater. Res..

[22] Wu Y.H., Yang H.J., Guo R.P., Wang X.J., Shi X.H., Liaw P.K., Qiao J.W. (2020). Tribological behavior of boronized Al_0.1_CoCrFeNi high-entropy alloys under dry and lubricated conditions. Wear.

[23] Yang R., Li F., Huang Z., Li Y., Qiao J. (2024). Tribological behavior of Fe_40_Mn_20_Cr_20_Ni_20_ HEA sliding against various counterface materials. Tribol. Int..

[24] Gain A.K., Li Z., Zhang L. (2023). Wear mechanism, subsurface structure and nanomechanical properties of additive manufactured Inconel nickel (IN718) alloy. Wear.

[25] Jiang X., Lu J., Zhao N., Chen Z., Zhao Z. (2024). A Review of Wear in Additive Manufacturing: Wear Mechanism, Materials, and Process. Lubricants.

[26] Zhao Y., Guo Q., Ma Z., Yu L. (2020). Comparative study on the microstructure evolution of selective laser melted and wrought IN718 superalloy during subsequent heat treatment process and its effect on mechanical properties. Mater. Sci. Eng. A.

[27] Choi J.P., Shin G.H., Yang S., Yang D.Y., Lee J.S., Brochu M., Yu J.H. (2017). Densification and microstructural investigation of Inconel 718 parts fabricated by selective laser melting. Powder Technol..

[28] Günen A. (2020). Properties and high temperature dry sliding wear behavior of boronized Inconel 718. Metall. Mater. Trans. A.

[29] Goel S., Ahlfons M., Bahbou F., Joshi S. (2018). Effect of different post-treatments on the microstructure of EBM-built alloy 718. J. Mater. Eng. Perform..

[30] Kirka M.M., Greeley D.A., Hawkins C., Dehoff R.R. (2017). Effect of anisotropy and texture on the low cycle fatigue behavior of Inconel 718 processed via electron beam melting. Int. J. Fatigue.

[31] Ivanov D., Travyanov A., Petrovskiy P., Cheverikin V., Alekseeva E., Khvan A., Logachev I. (2017). Evolution of structure and properties of the nickel-based alloy EP718 after the SLM growth and after different types of heat and mechanical treatment. Addit. Manuf..

[32] Qi H., Azer M., Ritter A. (2009). Studies of standard heat treatment effects on microstructure and mechanical properties of laser net shape manufactured INCONEL 718. Metall. Mater. Trans. A.

[33] Luo H., Cheng Z., Wang Y., Liu K., Xiang N., Jiang C. (2025). Microstructure, wear resistance and electrochemical properties of Inconel 718 alloy fabricated by laser powder bed fusion following different heat treatments. Mater. Today Commun..

[34] Song Y., Wang Q., Jing X., Xi Y., Dong L., Bai S. (2025). Unveiling localized protection and media-dependent degradation mechanism of Inconel 625 coating during tribocorrosion. Corros. Sci..

[35] Espallargas N., Mischler S. (2010). Tribocorrosion behaviour of overlay welded Ni–Cr 625 alloy in sulphuric and nitric acids: Electrochemical and chemical effects. Tribol. Int..

[36] Siddaiah A., Kasar A., Kumar P., Akram J., Misra M., Menezes P.L. (2021). Tribocorrosion behavior of Inconel 718 fabricated by laser powder bed fusion-based additive manufacturing. Coatings.

[37] Zhang Y., Niu X., Gao S., Wang R., Hou Y. (2026). Mechanical performance analysis of bolted angle connections exposure to pitting corrosion. Eng. Struct..

[38] Boinet M., Maximovitch S., Dalard F. (2003). Study of nickel passivation in a borate medium. J. Mater. Sci..

[39] Mischler S., Spiegel A., Stemp M., Landolt D. (2001). Influence of passivity on the tribocorrosion of carbon steel in aqueous solutions. Wear.

[40] Zhang Z., Ter-Ovanssian B., Norman B. (2021). Role of alloying elements in passive and transpassive behavior of Ni–Cr-based alloys in borate buffer solution. J. Electrochem. Soc..

[41] Kowalski M., Khurana A., Weil M., Munoz A.N.I., Mischler S. (2026). Effect of Hydrogen Charging on the Tribocorrosion Performance of C45CE Steel. Wear.

[42] Wood R.J.K., Lu P. (2024). Coatings and Surface Modification of Alloys for Tribo-Corrosion Applications. Coatings.

[43] (2004). Metallic Products—Types of Inspection Documents.

[44] (2026). Standard Specification for Precipitation-Hardening and Cold Worked Nickel Alloy Bars, Forgings, and Forging Stock for Moderate or High Temperature Service.

[45] Wieczorek D., Ulbrich D., Stachowiak A., Gruber K., Bartkowski D., Bartkowska A., Miklaszewski A. (2024). Heat treatment effects on tribocorrosion resistance of Inconel 718^®^ alloy produced by conventional and laser powder bed fusion methods. Int. J. Adv. Manuf. Technol..

[46] Zhao Y., Li K., Gargani M., Xiong W. (2020). A comparative analysis of Inconel 718 made by additive manufacturing and suction casting: Microstructure evolution in homogenization. Addit. Manuf..

[47] Newell D.J., O’Hara R.P., Cobb G.R., Palazotto A.N., Kirka M.M., Burggraf L.W., Hess J.A. (2019). Mitigation of scan strategy effects and material anisotropy through supersolvus annealing in LPBF IN718. Mater. Sci. Eng. A.

[48] Gruber K., Stopyra W., Kobiela K., Madejski B., Malicki M., Kurzynowski T. (2022). Mechanical properties of Inconel 718 additively manufactured by laser powder bed fusion after industrial high-temperature heat treatment. J. Manuf. Processes.

[49] López-Ortega A., Arana J.L., Bayón R. (2018). Tribocorrosion of Passive Materials: A Review on Test Procedures and Standards. Int. J. Corros..

[50] Feyzi M., Stack M.M., Hashemi R. (2024). Advancements in Modelling the Tribocorrosion Current. ChemElectroChem.

[51] Olsson C.-O.A., Igual Munoz A., Cao S., Mischler S. (2021). Modeling Current Transients in a Reciprocal Motion Tribocorrosion Experiment. J. Electrochem. Soc..

